# Point-of-Care Test for Detection of Urogenital Chlamydia in Women Shows Low Sensitivity. A Performance Evaluation Study in Two Clinics in Suriname

**DOI:** 10.1371/journal.pone.0032122

**Published:** 2012-02-29

**Authors:** Jannie J. van der Helm, Leslie O. A. Sabajo, Antoon W. Grunberg, Servaas A. Morré, Arjen G. C. L. Speksnijder, Henry J. C. de Vries

**Affiliations:** 1 STI Outpatient Clinic, Cluster Infectious Diseases, Public Health Service Amsterdam, Amsterdam, The Netherlands; 2 Dermatological Service, Ministry of Health, Paramaribo, Suriname; 3 Lobi Foundation, Paramaribo, Suriname; 4 VU University Medical Center, Amsterdam, The Netherlands; 5 Institute of Public Health Genomics, Department of Genetics and Cell Biology, Research Institutes CAPHRI and GROW, Faculty of Health, Medicine & Life Sciences, University of Maastricht, Maastricht, The Netherlands; 6 Public Health Laboratory, Cluster Infectious Diseases, Public Health Service Amsterdam, Amsterdam, The Netherlands; 7 Department of Dermatology, Academic Medical Center, University of Amsterdam, Amsterdam, The Netherlands; 8 Centre for Infections and Immunity Amsterdam, Academic Medical Center, University of Amsterdam, Amsterdam, The Netherlands; 9 Centre for Infectious Disease Control, National Institute of Public Health and the Environment, Bilthoven, The Netherlands; University of California San Francisco, University of California, Berkeley, and the Children's Hospital Oakland Research Institute, United States of America

## Abstract

**Background:**

In general, point-of-care (POC) tests for *Chlamydia trachomatis* (Ct) show disappointing test performance, especially disappointing sensitivity results. However, one study sponsored by the manufacturer (Diagnostics for the Real World) reported over 80% sensitivity with their Chlamydia Rapid Test (CRT). We evaluated the performance of this CRT in a non–manufacturer-sponsored trial.

**Methods:**

Between July 2009 and February 2010, we included samples from 912 women in both high- and low-risk clinics for sexually transmitted infections (STIs) in Paramaribo, Suriname. Sensitivity, specificity, positive- and negative predictive values (PPV and NPV) for CRT compared to NAAT (Aptima, Gen-Probe) were determined. Quantitative Ct load and human cell load were determined in all CRT and/or NAAT positive samples.

**Results:**

CRT compared to NAAT showed a sensitivity and specificity of 41.2% (95% CI, 31.9%–50.9%) and 96.4% (95% CI, 95.0%–97.5%), respectively. PPV and NPV were 59.2% (95% CI, 47.5%–70.1%) and 92.9% (95% CI, 91.0%–94.5%), respectively. Quantitative Ct bacterial load was 73 times higher in NAAT-positive/CRT-positive samples compared to NAAT-positive/CRT-negative samples (p<0.001). Human cell load did not differ between true-positive and false-negative CRT results (p = 0.835). Sensitivity of CRT in samples with low Ct load was 12.5% (95% CI, 5.2%–24.2%) and in samples with high Ct load 73.5% (95% CI, 59.9%–84.4%).

**Conclusions:**

The sensitivity of CRT for detecting urogenital Ct in this non–manufacturer-sponsored study did not meet the expectations as described previously. The CRT missed samples with a low Ct load. Improved POC are needed as meaningful diagnostic to reduce the disease burden of Ct.

## Introduction

Urogenital chlamydia is the most prevalent, curable bacterial sexually transmitted infection (STI) worldwide [1], with a significant public health burden, especially in young women [2]. The causative bacterium, *Chlamydia trachomatis* (Ct) causes a high rate of asymptomatic infections [3] and is associated with adverse outcomes like infertility, ectopic pregnancy and pelvic inflammatory disease (PID) [4]. To reduce transmission and late complications, active case finding and early treatment are critical strategies. The standard diagnostics are Nucleic Acid Amplification Tests (NAAT), but they are expensive and require sophisticated laboratory conditions [5]. This makes NAAT unsuitable for the detection of Ct for most low-resource settings [6]. Therefore the World Health organization (WHO) has launched a priority program that is designated to develop affordable and reliable point-of-care (POC) tests for STIs that are predominant in low resource countries [http://www.who.int/std_diagnostics]. In this program, WHO has formulated the ASSURED criteria that POC tests have to meet: Affordable, Sensitive, Specific, User-friendly, Robust and rapid, Equipment-free, Deliverable to those who need them [7]. The POC test result should be readily available, while the patient waits, to ensure prompt treatment. This is especially important where patient return for treatment is low. It is estimated that a POC test of moderate sensitivity (63%) combined with immediate treatment on-site may lead to the treatment of more infected individuals than an ultra-sensitive and specific NAAT alone when patient return is low [8]. Moreover, counselling messages are most efficient when a diagnosis can be communicated during the same consultation [9]. These advantages are relevant for industrialized countries as well, even if POC tests have a lower sensitivity than standard NAAT.

To date, POC tests for urogenital chlamydia show disappointing test characteristics, especially low sensitivity. In a recent evaluation, three POC tests for urogenital chlamydia, currently on the market, showed poor sensitivity between 12% and 17% in a non–manufacturer-sponsored clinical study [10]. In contrast, one POC test for urogenital chlamydia (Diagnostics for the Real World, Cambridge, UK) especially developed for low-resource countries has an asserted sensitivity of over 80% [11]. A manufacturer-sponsored diagnostic field study in the Philippines revealed sensitivities of 71% and 87% among women at high risk and low risk for STI, respectively [12]. Suriname, South America, is a low-resource country and affordable and reliable diagnostics to detect Ct are urgently needed. Therefore, we aimed to evaluate the performance of this promising POC test in two outpatient clinics in Suriname, with the objective to use this test for intervention of the chlamydia epidemic.

## Methods

### Study sites and population

The study was approved by the ethical committee of the Ministry of Health of the Republic of Suriname (VG010-2007) and the ethical committee of the Academic Medical Centre, University of Amsterdam, the Netherlands (MEC07/127). Patients were recruited at two sites in Paramaribo, Suriname:

1. The Dermatological Service, an integrated outpatient clinic that offers free-of-charge examination and treatment of STIs and infectious skin diseases like leprosy and leishmaniasis. All consecutive women who visited for an STI check-up were asked to participate in the study and were considered to be at high-risk for STI.
2. The Lobi Foundation is a center for birth control and sexual health. As women who visit this clinic do not attend primarily to be checked for STI, these participants were considered to be at low risk for STI.

Recruitment took place between July 2009 and February 2010. Exclusion criteria were: use of antibiotics in the past 7 days, age younger than 18 years and previous participation. After written informed consent, patients were given a unique code to participate anonymously. Participants were interviewed about demographic characteristics, including self-reported ethnicity as Suriname is a multiethnic society, with many ethnic groups such as Creoles and Maroons (both descendants from the African diaspora due to slave trade), Hindustani, Javanese, and Chinese (all descendants from labor immigrants), Caucasians (descendants from Dutch farmers), indigenous Amerindian people and Mixed race persons. Moreover, participants were asked about willingness to wait for POC test results, although in our study participants did not receive the results from POC, and if they used any products for vaginal hygiene like douches, herbs, or other home products, and if so, in what frequency. Data were entered into an MS Access database.

### Specimen collection and testing procedures

Nurse-collected vaginal swabs were obtained blindly for the Chlamydia Rapid Test (CRT) (Diagnostics for the Real World (Europe), Cambridge, UK) and NAAT (Aptima, Gen-Probe, San Diego, USA) testing using a cross-over model. This means that in the first half of the included women the swab for the CRT was taken first and the second of the included women NAAT was taken first. Nurses were trained to collect the swabs before routine speculum examination was performed. A minimum period of 10 times for CRT and 10 seconds for NAAT of contact between the tip of the swab and the vaginal wall in a rotating motion was ensured. CRT was immediately performed according to the manufacturer's instructions on-site in the laboratory. All technicians that performed the CRT were trained with proficiency panels as provided and instructed by the manufacturer. Technicians did not receive information about the participant. The test results were interpreted and recorded by two laboratory technicians separately. CRT results were defined as indeterminate when the laboratory technicians reported discordant results or when CRT failed (i.e. control line did not appear). The samples for NAAT testing were collected according to the manufacturer's instructions, and shipped to the Public Health Laboratory in Amsterdam where they were tested within 50 days after collection. NAAT test results were communicated with the two recruitment sites in Suriname and participants with a positive-Ct NAAT were treated with doxycycline 100 mg bid for 7 days at Lobi Foundation and 10 days bid at the Dermatological Service or, in case of (possible) pregnancy, with a single 1000 mg oral dose of azithromycin.

### Chlamydia Rapid Test

The CRT was performed as described previously [13]. Version 6.1 of the *Chlamydia* Rapid Test (Professional use) (P/N 1200-20) instructions for use (C03-0008) was used. Shortly, each swab was subjected to extraction by sequential addition of 400 µl of reagent 1, 300 µl of reagent 2, and 100 µl of reagent 3 to the swab in a tapered sample preparation tube, with gentle mixing between additions. The sample preparation reagents were administered with unit dose pipettes. The extraction tube was then capped and used as a dropper to deliver 5 drops (approximately 100 µl) of the extracted sample to a tube containing the lyophilized amplification and detection reagents. The resulting mixture was agitated gently until a clear pink solution was obtained, after which the test strip, coated with a monoclonal antibody to chlamydial lipopolysaccharide (LPS) and including a procedural control, was added to the solution and allowed to stand for 25 minutes before the result was read. Each swab was subjected to one extraction. The test strip was used in the interpretation of the result; a clearly visible test line indicated a positive result, provided that the control line was also visible on the test strip.

### NAAT testing

For NAAT testing, the monospecific Aptima chlamydia assay for the detection of *Chlamydia trachomatis* rRNA (Gen-Probe Inc., San Diego, USA) was used with the accompanying vaginal swab specimen collection kit. The protocols described in the package inserts were followed. Technicians performing NAAT were blinded to the results of the POC-Ct and did not receive clinical information. This NAAT is an FDA-approved commercial test and was used to estimate the Ct prevalence at both study sites.

### Quantitation of Ct load and HLA

Quantitative Ct load was determined for samples with a discrepant test result between CRT and NAAT, and for samples that tested positive for CRT as well as for NAAT using a real-time PCR targeting the cryptic plasmid [14]. Ct load was expressed as inclusion forming units (IFU) based on defined serial dilutions of Ct cultured in human cells with over >90% infected HeLa cells of 100 IFU to 0.001 IFU taking into account also DNA from non-viable Ct particles. The human cell load was assessed by determination of human HLA copies in combination with a defined serial dilution of quantified human DNA using the following primer probe combination: HLA-F 5′-TTG-TAC-CAG-TTT-TAC-GGT-CCC-3′ HLA-R 5′- TGG-TAG-CAG-CGG- TAG-AGT-TG,-3 and HLA-Probe 5′-FAM- TTC TAC GTG GAC CTG GAG AGG AAG GAG -BHQ1-3′. By using a chlamydial and a human target, the average chlamydial/human cell ratio, and IFU/swab were calculated [10].

### Statistical analysis

To evaluate the performance of CRT compared to NAAT sensitivity, specificity, positive predictive value (PPV) and negative predictive value (NPV) were calculated using standard methods. Specimens with indeterminate results by CRT were excluded. An independent t-test was used to compare log-transformed Ct loads between true-positive and false-negative CRT results. Analyses were performed with SPSS package version 19.0 (SPSS Inc., Chicago, IL).

The study has been reported according to the STARD checklist for the reporting of studies of diagnostic accuracy.

## Results

### Study population and specimens

**Figure 1:**
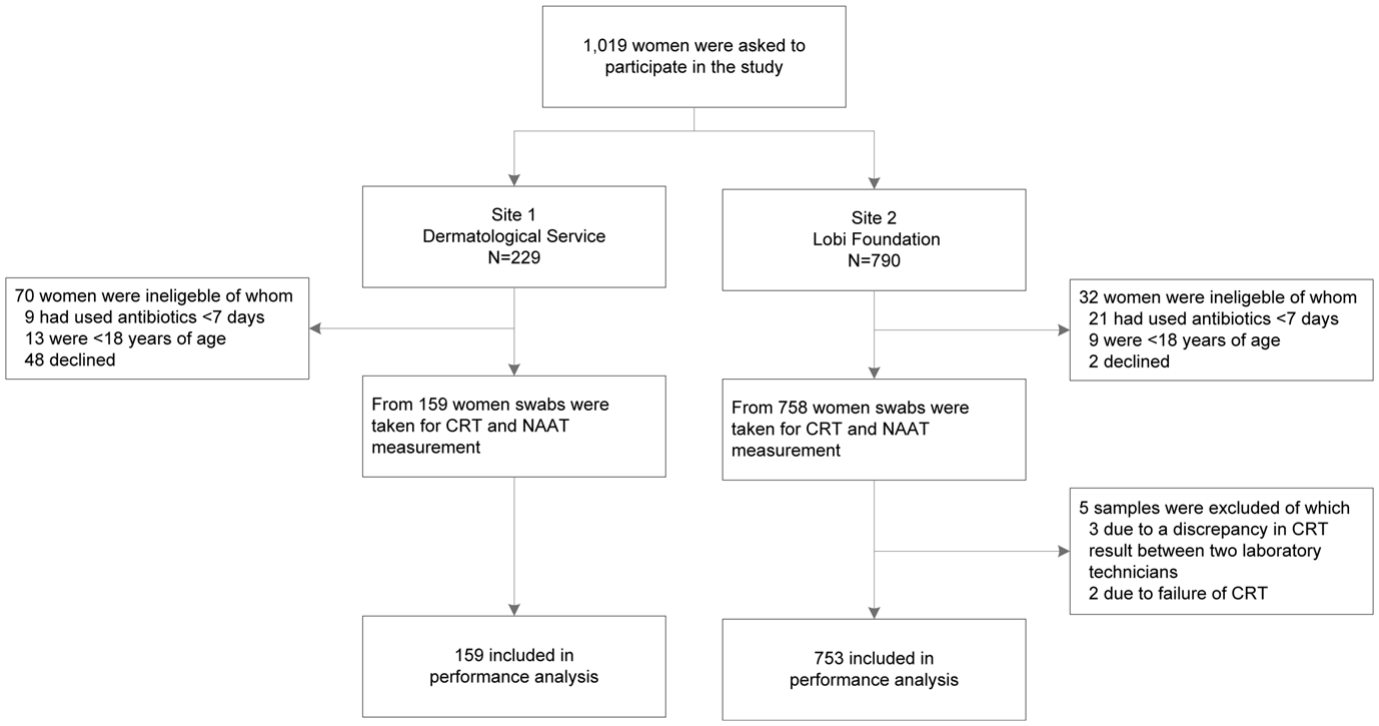
Flow chart of specimen collection for the evaluation of a Chlamydia Rapid Test test as diagnostic for urogenital chlamydia in women at two study sites in Paramaribo, Suriname, from July 2009 to February 2010. NAAT; Aptima chlamydia single test, Genprobe (control test) CRT; Chlamydia Rapid Test, Diagnostics for the Real World (evaluated test).

In total, 1019 women were asked to participate in the study, of whom 917 were included and 102 did not meet the inclusion criteria or declined to participate (Figure 1). Five women were excluded from the CRT performance evaluation due to either discrepancy in CRT result between two lab technicians (n = 3) or failure of CRT (n = 2).

General characteristics of the 912 women included in the CRT performance evaluation are shown in Table 1. Their median age was 30 years (IQR 25–36), 336 (36.9%) were of Creole/Maroon ethnicity and 229 (25.1%) were of Hindustani ethnicity. Twenty-one (2.3%) women reported having had sex for money or goods. Almost all women 900 (98.7%) would wait for the CRT test result if the test were a standard offering in their clinic. Of these women, 660 (73.3%) would be willing to wait for a maximum of half an hour to receive the results, the other 240 (26.7%) would be willing to wait for at least an hour.

**Table 1:** General characteristics of the 912 women included in the evaluation of a Chlamydia Rapid Test as diagnostic for urogenital chlamydia in women at two study sites in Paramaribo, Suriname, from July 2009 to February 2010.

	Dermatological Service (n = 159)	Lobi Foundation (n = 753)	Total population (n = 912)
	N (%)	N (%)	N (%)
Median age in years (IQR)	27 (22–34)	30 (25–37)	30 (25–36)
Ethnic Group			
Caucasian	5 (3.1)	6 (0.8)	11 (1.2)
Chinese	1 (0.6)	5 (0.7)	6 (0.7)
Creole/Maroon	81 (51.0)	255 (33.9)	336 (36.9)
Hindustani	17 (10.7)	212 (28.2)	229 (25.1)
Indigenous Amerindian	5 (3.1)	9 (1.2)	14 (1.5)
Javanese	10 (6.3)	137 (18.2)	147 (16.1)
Mixed race	39 (24.5)	127 (16.9)	166 (18.2)
Sex for money or goods			
Yes	17 (10.7)	4 (0.5)	21 (2.3)
No	140 (88.1)	735 (96.7)	875 (95.9)
Unknown	2 (1.3)	14 (1.9)	16 (1.7)
Willing to wait for POC-Ct test result	159 (100)	741 (98.4)	900 (98.7)
Maximum time these women are willing to wait			
Half an hour	84 (52.8)	576 (77.7)	660 (73.3)
At least an hour	75 (47.2)	165 (22.3)	240 (26.7)
Symptoms			
Dysuria	56 (35.2)	173 (23.0)	229 (25.1)
Irregular menstruation	53 (33.0)	194 (25.8)	247 (27.1)
Lower abdominal pain	72 (45.3)	302 (40.1)	374 (41.0)
Pain during intercourse	40 (25.2)	192 (25.5)	232 (25.4)
Vaginal discharge	110 (69.2)	369 (49.0)	479 (52.5)
Use of any vaginal cleansing			
Yes	80 (50.3)	228 (30.3)	308 (33.8)
No	74 (46.5)	512 (68.0)	586 (64.3)
Unknown	5 (3.1)	13 (1.7)	18 (2.0)
Frequency of cleansing among those who practice vaginal cleansing			
At least once a week	43 (53.8)	110 (48.2)	153 (49.7)
Less than once a week	18 (22.5)	56 (24.6)	74 (24.0)
Unknown	19 (23.8)	62 (27.2)	81 (26.3)

IQR; interquartile range.

### Ct prevalence and CRT performance results

Ct prevalence was 20.8% in the high-risk population (visiting the Dermatological Service) and 9.2% in the low-risk population (visiting Lobi Foundation). Combining the results of the two sites, the sensitivity and specificity of the CRT in identifying Ct compared to NAAT were 41.2% (95% CI, 31.9%–50.9%) and 96.4% (95% CI, 95.0%–97.5%), respectively. PPV of the CRT was 59.2% (95% CI, 47.5%–70.1%) and NPV was 92.9% (95% CI, 91.0%–94.5%). Sensitivity and specificity of CRT compared to NAAT were comparable for the high-risk population (39.4% and 94.4%) and the low-risk population (42.0% and 96.8%) (Table 2).

**Table 2:** Performance results of the Diagnostics for the Real World Chlamydia Rapid Test (CRT) compared to NAAT (Aptima chlamydia single test).

	Sensitivity (%), 95% CI	Specificity (%), 95% CI	PPV (%), 95% CI	NPV (%), 95% CI
Total population (n = 912)	41.2% (42/102), 31.9%–50.9%	96.4% (781/810), 95.0%–97.5%	59.2% (42/71), 47.5%–70.1%	92.9% (781/841), 91.0%–94.5%
Dermatological Service (n = 159)	39.4% (13/33), 24.0%–56.6%	94.4 (119/126), 89.3–97.5	65.0 (13/20), 42.7–83.2	85.6 (119/139), 79.0–90.7
Lobi Foundation (n = 753)	42.0 (29/69), 30.8–53.9	96.8 (662/684), 95.3–97.9	56.9 (29/51), 43.1–69.9	94.3 (662/702), 92.4–95.8

Evaluation of a CRT as diagnostic for urogenital chlamydia in women at two study sites in Paramaribo, Suriname, from July 2009 to February 2010.

PPV; positive predictive value.

NPV; negative predictive value.

95% CI; 95% confidence interval.

### Quantitative load measurements

Quantitative Ct bacterial load and human HLA were assessed for the samples that showed discrepant results for CRT and NAAT (n = 89) and for samples that were CRT and NAAT positive (n = 42). Ct bacterial load could be detected in 99/131 samples and human HLA in 126/131 samples. Of the 42 samples that tested positive for CRT and NAAT, quantitative Ct bacterial load was detected in all 42 samples and human HLA in 39 samples. Of the 60 samples that tested CRT negative and NAAT positive, quantitative Ct bacterial load was detected in 55 samples and human HLA in all 60 samples. Of the 29 samples that tested CRT positive and NAAT negative, quantitative Ct bacterial load was detected in 2 samples and human HLA in 27 samples (Table 3).

**Table 3:** *C. trachomatis* quantitative bacterial load and human cell load measurements in concordant and discordant samples with NAAT (Aptima chlamydia single test) and the Diagnostics for the Real World Chlamydia Rapid Test (CRT).

	NAAT+/CRT+ (n = 42)	NAAT+/CRT (n = 60)	P-value	NAAT−/CRT+ (n = 29)
Quantitative Ct load assessed	42	55		2
Geometric mean Ct load (IFU)	119.6	1.6	<0.001	
Range (IFU)	5.57–6470	0.0061–519		0.00261 and 62.9
Quantitative human cell load	39	60		27
Geometric mean human cell load (HLA copy)	344.0	326.7	0.835	451.43
Range human cell load (HLA copy)	5.27–1460	7.99–1930		8.01–7800
Concentration Ct load per human cell assessed (IFU/HLA copy)	39	55		2
Geometric mean of concentration (IFU/HLA copy)	0.32	0.0053	0.001	
Range (IFU/HLA copy)	0.0079–107.2	3.9*10^−6^ – 1.87		1.5*10^−6^ and 0.15

Evaluation of a CRT as diagnostic for urogenital chlamydia in women at two study sites in Paramaribo, Suriname, from July 2009 to February 2010.

IFU; inclusion forming units.

HLA; human leucocyte antigen gene.

Quantitative Ct bacterial load was 73 times higher in NAAT-positive/CRT-positive samples (geometric mean 120 IFU) compared to NAAT-positive/CRT-negative samples (geometric mean 1.64 IFU, p<0.001). Human DNA concentration did not differ between the true-positive and false-negative CRT results (p = 0.835). The average chlamydial/human cell load ratio (Ct concentration) was 60 times higher in NAAT-positive samples where CRT detected Ct infection (geometric mean 0.32 IFU/human cell) compared to loads that CRT did not detect (geometric mean 0.0053 IFU/human cell, p<0.001). Quantitative HLA load was comparable for NAAT-positive/CRT-positive samples (geometric mean 344 cells) compared to NAAT-negative/CRT-positive samples (geometric mean 451 cells, p = 0.424).

Quantitative Ct loads were comparable for women reporting symptoms like vaginal discharge, irregular menstruation, pain during intercourse, lower abdominal pain or dysuria and women without the specific symptom (data not shown). Women visiting the high-risk STI clinic had comparable quantitative Ct loads with those visiting the low-risk clinic (p = 0.525). Sensitivity of the CRT was comparable for those who practiced any vaginal hygienic measures, 37.5% (95% CI, 23.6%–53.1%), compared to those who did not practice vaginal cleansing, 43.3% (95% CI, 31.3%–56.0%). When comparing women who practice vaginal cleansing frequently, at least once a week, with those who cleanse less than once weekly, sensitivity of CRT yields comparable results, 39.1% (95% CI, 21.1%–59.8%) and 27.3% (95% CI, 7.5%–57.8%), respectively.

Based on the overall median Ct load, NAAT-positive samples were divided in two groups with either a low- (range 0.006–12.5 IFU) or high-grade quantitative bacterial Ct load (range 13.5–6470 IFU). In the low-grade bacterial load group, the CRT sensitivity was 12.5% (95% CI, 5.2%–24.2%), whereas in the high-grade Ct load group the sensitivity was 73.5% (95% CI, 59.9%–84.4%).

## Discussion

We found a disappointingly low clinical sensitivity of 42.0% and 39.4% of the CRT in low-risk and high-risk Surinamese women, respectively, compared to the sensitivity of 86.8% in low-risk women and 71% in high-risk women in the Philippines, reported earlier in a study supported by the manufacturer [12]. The discrepancy might partly be explained by the use of a different reference test. Where we used Gen-Probe's Aptima platform as a reference test, in the Philippines study the Roche Amplicor (Roche Molecular Systems, Branchburg, NJ) was used. Although current generation NAATs have comparable sensitivities, NAAT could be more sensitive than Roche Amplicor [15]. A somewhat lower sensitivity of CRT in our study could be expected with a more sensitive control test, but this does not explain the large difference in sensitivity found in the Philippine study and our results.

Another explanation for the lower sensitivity we found could be attributed to a different wash-out period for antibiotic use between the two studies. We excluded women who used antibiotics in the last 7 days, while in the Philippines study women who used antibiotics in the previous month were excluded. Time to clearance of LPS antigen, which is targeted by the CRT, might be shorter after antibiotic use than time to clearance of Ct rRNA, which is targeted by NAAT [16]. This could have caused the occurrence of false-positive NAAT samples, and consequently more false-negative CRT samples could be expected. Low sensitivity of the CRT due to inadequate collection resulting in a low sample yield could be ruled out since the human cell load in samples with true-positive and false-negative CRT results was comparable. The CRT had a 96.4% specificity. False-positive CRT results could have been caused by cross reactivity with *C. ptsittaci* or *C. pneumoniae* as described in the manufacturers manual. Yet infections with these organisms in the urogenital tract in humans are uncommon [17], [18]. As a false positive chlamydia diagnosis can have serious adverse social consequences a specificity of 96,4% is undesirable, especially in low prevalent settings. The CRT in our study had a few modifications compared to the study in the Philippines. We used unit dose pipettes instead of unit dose vials. Also, the nitrocellulose membrane was changed by the manufacturer and according to the manual, only one dipstick had to be used to interpret the results. However, when a test is renewed one might expect at least comparable diagnostic characteristics compared to the previous test.

In the CRT evaluation study performed in the Philippines, the Ct prevalence was 6.3% in the low-risk group (women visiting an obstetrics-gynaecology clinic) and 17.9 to 32% in the high-risk group (female sex workers), which compares well with the prevalences found in our study, 9.2% and 20.8% respectively. The sensitivity figures found in our study were comparable for low-risk and high-risk women, 42.0% and 39.4% respectively. Quite surprisingly, in the Philippines study a much lower sensitivity was found in the high-risk group compared to the low-risk group. The authors explain this finding as a result of the use of vaginal creams and other feminine hygiene products, which can interfere with the CRT. In our study, the sensitivity of CRT was comparable for women who practiced any vaginal cleansing and those who did not.

Although we consider the population recruited at Lobi Foundation a low risk group, with a prevalence of 9.2% this population would be considered high risk in many settings. Yet, with a prevalence of 20.8% as found at the Dermatological Service, the difference in prevalence between the two study sites is substantial.

The sensitivity of CRT is higher in samples with a high bacterial load. The clinical relevance of organism load is still debated, but it is suggested that infections with high organism loads are more likely to lead to cervicitis or PID and are associated with multiple patient-reported symptoms [19]. However, the association with patient-reported symptoms was only found with first-void urine and endocervical samples and not with self-collected vaginal samples. In our study, where nurse-collected vaginal swabs were used, quantitative Ct loads were not significantly different for asymptomatic women and women reporting one or multiple symptoms such as vaginal discharge or dysuria.

The NAAT platform is a latest generation highly sensitive commercial diagnostic test for Ct [20]. However no test is 100% accurate and a positive bacterial Ct load signal was detected in two samples that were NAAT negative and CRT positive. One sample had a Ct load of 62.9 IFU which might be explained by inhibition of high target load [21]. The other sample had a very low load of 0.00261 IFU. Since the frequency of these discrepancies was extremely low, we do not consider that this finding significantly affects our test evaluation.

A recent field study of the same CRT test but to detect ocular chlamydia infection (trachoma) found similar disappointingly low sensitivity (33.3%–67.9%) and specificity (92.4%–99.0%) [22]. Most commercially available and clinically evaluated POC tests for urogenital chlamydia show poor sensitivity results [10]. Compared with the results found in our evaluation, the CRT of Diagnostics for the Real World outperforms some of the other commercially available products [10]. Still, with a sensitivity of only 41.7%, this test performs under the minimally required sensitivity of 63% required for a POC test to treat more infected individuals than the standard NAAT in a setting with low patient return (<65%), [8]. On the other hand, in situations where transmission during treatment delay and low return for treatment are considerable, even a POC test with a sensitivity below 63% could be beneficial in the prevention of ongoing STI transmission [23]. A recent economic evaluation analysis using the same CRT as we evaluated in this study, showed that in the UK using NAAT is more cost-effective. [24]. In that evaluation, a sensitivity between 73% and 85% for the CRT was assumed.

POC tests available for systemic infections like HIV and syphilis are highly sensitive since they are based on the detection of serum antibodies [25], [26]. Infections caused by organisms like Ct (but also *N. gonorrhoeae)* are confined to mucosal tissue and normally invoke little to no production of antibodies. Therefore, the development of POC tests to diagnose mucosal Ct infections based on the detection of serum antibodies is, at least for now, not an option. Improved POC tests for Ct need to detect bacterial antigens or nucleic acids, even in cases with a low bacterial load. Promising steps have been made in the field of POC HIV-load NAAT using nanotechnology [27]. Along the same lines, a POC test for urogenital chlamydia with sufficient sensitivity could be developed. Until reliable and affordable diagnostics are available, algorithms for syndromic management can be used for low-resource settings, although the success of algorithms for vaginal discharge varies between populations [28].

In conclusion, the evaluated CRT of Diagnostics for the Real World has no added value in the management of Ct infections due to its low test performance. There is an urgent need for POC diagnostics for the detection of urogenital chlamydia meeting the ASSURED criteria, including adequate sensitivity.
